# A Study on the Impact of Integrating Reinforcement Learning for Channel Prediction and Power Allocation Scheme in MISO-NOMA System

**DOI:** 10.3390/s23031383

**Published:** 2023-01-26

**Authors:** Mohamed Gaballa, Maysam Abbod, Ammar Aldallal

**Affiliations:** 1 Department of Electronic & Electrical Engineering, Brunel University London, Uxbridge UB8 3PH, UK; 2 Department of Telecommunication Engineering, Ahlia University, Manama 10878, Bahrain

**Keywords:** RL, *Q*-learning, MISO-NOMA, KKT conditions

## Abstract

In this study, the influence of adopting Reinforcement Learning (RL) to predict the channel parameters for user devices in a Power Domain Multi-Input Single-Output Non-Orthogonal Multiple Access (MISO-NOMA) system is inspected. In the channel prediction-based RL approach, the Q-learning algorithm is developed and incorporated into the NOMA system so that the developed Q-model can be employed to predict the channel coefficients for every user device. The purpose of adopting the developed Q-learning procedure is to maximize the received downlink sum-rate and decrease the estimation loss. To satisfy this aim, the developed Q-algorithm is initialized using different channel statistics and then the algorithm is updated based on the interaction with the environment in order to approximate the channel coefficients for each device. The predicted parameters are utilized at the receiver side to recover the desired data. Furthermore, based on maximizing the sum-rate of the examined user devices, the power factors for each user can be deduced analytically to allocate the optimal power factor for every user device in the system. In addition, this work inspects how the channel prediction based on the developed Q-learning model, and the power allocation policy, can both be incorporated for the purpose of multiuser recognition in the examined MISO-NOMA system. Simulation results, based on several performance metrics, have demonstrated that the developed Q-learning algorithm can be a competitive algorithm for channel estimation when compared to different benchmark schemes such as deep learning-based long short-term memory (LSTM), RL based actor-critic algorithm, RL based state-action-reward-state-action (SARSA) algorithm, and standard channel estimation scheme based on minimum mean square error procedure.

## 1. Introduction

The Non-Orthogonal Multiple Access (NOMA) system has been characterized as an inspiring multiple access form for upcoming wireless approaches to enhance the spectral efficiency and throughput [1]. NOMA system can develop the available resources more realistically by efficiently, taking into consideration the users’ channel environments and also giving support to several users with distinctive Quality of Service (QoS) needs [2]. The integration of NOMA and multiple antenna techniques can be exploited to improve and reinforce system performance [3], therefore, inspecting Multiple Input-Single Output (MISO) NOMA system can be a good example in the direction of characterizing the expected upgrade in achievable data rates [4]. In downlink NOMA structure, the receiver device can receive a multiplexing of signals transmitted to several user terminals in the NOMA cell, thus eliminating the interference generated by other user devices come to be essential for coordinated detection. Frequently in power domain NOMA (PD-NOMA), multiuser detection can be handled via successive interference cancellation (SIC) [5]. In the SIC procedure, symbols from numerous users are decoded successively on the basis of the Channel State Information (CSI) and power percentage designated for each user. A broad investigation of CSI for various users is demanding because pilot data that can be exploited in channel prediction, might interfere with symbols from other user terminals, therefore affecting the performance of a conventional prediction scheme, such as the Minimum Mean Square Error (MMSE) estimator [6]. Furthermore, power allocation policy is considered an essential issue for user devices when PD-NOMA is considered [7].

Deep Learning (DL) or Reinforcement Learning (RL) techniques, have the ability to track the differences in the channels among users and BS, thus, they are recently considered a powerful tool for upcoming radio systems [8,9]. Hence, allocating the power factors or estimating the CSI for user devices with the assistance of Machine Learning (ML) algorithms, triggered the authors for more deep investigations into this field in order to enhance the performance and detection process.

### 1.1. Related Works

Different techniques were introduced by authors in [10] to realize the optimal MMSE channel estimator in the Reconfigurable Intelligent Surfaces (RIS)-based MISO system. In the first technique, the authors suggest an analytical linear estimator to adjust the phase shift matrix of the RIS during the training phase, and the estimator based on that technique is shown to produce sensible accuracy compared to the least-squares method when the statistical properties of the applied channel and noise are considered. In the other approach, authors have expressed the channel prediction problem as an image denoising problem, then they introduce a Convolutional Neural Network (CNN) to achieve the denoising and predict the optimal MMSE channel parameters. Numerical outcomes have clarified that the proposed estimator based CNN algorithm can offer improved performance compared to the linear estimation method and low computational intricacy is preserved.

Toward enhancing the link reliability, a neural network model for a wireless channel estimator is proposed in [11] to be used with uncoded space-time diversity procedure in Multi Input Multi Output (MIMO) system. Based on the neural network ML structure, a channel estimator is suggested, and a mathematical scheme is presented to derive an optimum power transmission factors that can assist in lessening the channel prediction bandwidth utilization. Simulation results revealed that the channel estimator based on the proposed neural network structure can deliver an improvement in Bit Error Rate (BER) and Mean Square Error (MSE) compared to the standard MMSE channel estimation technique.

In a massive MIMO system and on the basis of a deep autoencoder scheme, authors in [12] performed experimental verifications on two tasks, one task for channel estimation modelling for wireless links, and the other task is belonging to a power allocation policy. The proposed deep learning autoencoder is also used to manage the issue raised from inadequate training datasets that may cause critical overfitting problems and consequently affect the model’s reliability. Results based on the autoencoder procedure clarified that the suggested scheme could successfully enhance performance when the extent of the training dataset is mainly within a specified threshold selection.

To get over limitations raised when standard iterative power control techniques are utilized, such as high complexity and unnecessary latency, the work in [13] introduced a deep learning framework to manage these issues. In the presented structure, the outdated and partial CSI is exploited, and a Deep Neural Network (DNN) framework is created to construct an optimization problem to boost the spectral efficiency in device-to-device communication systems. User fairness and energy efficiency constraints were examined, and simulation outcomes showed that the proposed DNN model can attain better spectral and energy efficiency compared to the MMSE procedure when numerous channel correlation factors are considered.

Based on CSI, the position of each user device with respect to BS, and the path loss, a deep learning framework labelled PowerNet is introduced in [14]. The authors attempt to prove that it is possible to avoid the time consumption involved with intricate channel estimation procedures, and at the same time, power control can be managed. Different from traditional DNNs that employ a fully connected structure, the presented PowerNet method utilizes a CNN layers to recognize the interference model through several links in wireless networks. Simulation outcomes revealed that the suggested PowerNet scheme can realize a stable performance without explicit channel estimation.

Recently, approximating the channel parameters or predicting the power factors with the assistance of Reinforcement learning (RL), is investigated by many researchers. The authors of [15] proposed an end-to-end channel estimation framework for a downlink multiuser multiple antenna system. The authors presented an RL-based actor-critic scheme for channel estimation without the assumption of ideal CSI. The authors mainly depend on the agent to bring and utilize the pilot symbols into the estimation process and then employ the estimated channel parameters to create downlink beamforming matrices. To satisfy the purpose of maximizing the sum rate reward, network parameters are adjusted based on the deep policy gradient method. The results proved that the suggested channel estimation algorithm can provide convergence and stable performance under various channel statistics and can perform better than the typical MMSE procedure when the sum rate metric is examined.

In [16], the authors developed a Deep Reinforcement Learning (DRL) method for device-to-device pairing to understand the correlation patterns between wireless networks. The introduced RL algorithm is adopted to explore the joint channel selection and power control problem for device-to-device pairing and to boost the weighted sum rate. Based on the suggested DRL learning procedure, each device-to-device pair can make use of the outdated and local information to understand the network parameters and perform decisions independently. Results showed that without a global CSI, the suggested DRL scheme is capable to attain a stable performance close to that achieved using standard analytical approaches.

The combination between a DNN as a tool for channel prediction and an optimized power scheme is explored in [17] for the purpose of multiuser detection in the NOMA system. The DNN based Long Short-Term Memory (LSTM) network is developed for channel prediction based on complex data processing. The DNN network is trained on the basis of both the correlation between successive training sequences and the normalised channel statistics. The efficiency of the suggested DNN based LSTM for channel prediction is inspected using different fading models and simulation outcomes, in terms of different performance metrics, have proved that the presented DNN scheme for channel estimation can provide a consistent performance compared to the MMSE procedure even when cell capacity is expanded.

### 1.2. Research Gap and Significance

Based on the preceding works, many of the proposed schemes that consider predicting the channel parameters task are mainly focused on implementing several deep neural networks (DNN) while applying RL approaches, which in turn leads to an increase in the number of hidden layers with a massive number of neurons in each layer. The significance of this study is to illuminate that we can eliminate the need for such DNN approaches, and instead, we can adopt the RL based developed Q-learning algorithm to predict the channel coefficients for each user device in MISO-NOMA cell, and at the same time, a notable improvement in system performance and network convergence is realized. The most prominent gain of the developed channel estimator scheme is that it can enhance the system performance without the need for hidden layers or an external training set.

In addition, several RL algorithms have been proposed to explicitly address the issues associated with channel state information (CSI), beamforming, and power allocation. To the best of the authors’ knowledge, there is no study that explores the incorporation between Q-learning algorithm for channel prediction and the power allocation policy as an integrated scheme for multiuser detection in downlink MISO-NOMA system in fading channels.

Furthermore, it is worth mentioning that unlike deep learning algorithms, that mainly depend on learning from a training data set, the proposed Q-learning algorithm in our study is developed to dynamically enhance the system performance and adjust to the variations in the channel based on the feedback from the environment.

### 1.3. Contributions to Knowledge

The channel prediction problem in downlink NOMA systems was considered in numerous works. In addition, there have been several works that apply machine learning (ML) to handle the channel estimation task in wireless communication systems. However, most of the current research on channel prediction in the NOMA systems based on ML is introduced via deep neural networks. To the best of the author’s knowledge, currently, there is no research that manages the channel approximation task in a multiuser multi-input single-output NOMA system through an RL based Q-learning algorithm. The RL based Q-learning algorithm is developed based on maximizing the sum rates for all users in the network such that it can be used efficiently to predict the channel parameters for each user in the MISO-NOMA cell.

In addition, in this work, a structured mathematical analysis is introduced to formulate a non-complex analytical form for the power allocation for user devices in the examined MISO-NOMA system based on boosting the sum rate of the system while considering the constraints of the total power budget in the system, and the QoS for each user. Furthermore, the performance of the MISO-NOMA system is investigated when both the developed Q-learning algorithm for channel estimation and the derived power allocation scheme are jointly implemented. In this work, the contributions can be summed up as shown:

- In this study, a framework is proposed to illuminate how RL based Q-learning algorithm is developed based on maximizing the sum rates for all users in a MISO-NOMA system in order that it can be used dynamically to predict the channel parameters for each user in the MISO-NOMA cell.
- As a reference comparison, four further simulation environments are established. (1) the standard minimum mean square error (MMSE) based channel prediction scheme (Neumann et al.); (2) the DNN algorithm based on LSTM network for channel prediction applied in [17], (3) the RL based actor-critic procedure for channel prediction applied in [15], (4) the fourth simulation environment is dependent on applying RL based State-Action-Reward-State-Action (SARSA) procedure (Ahsan et al. and Mu et al.). The simulation outcomes of these environments are compared with the results of our proposed RL based Q-learning scheme, and the results emphasized that dependability can be assured by our developed Q-model for predicting channel parameters even when the number of devices in the cell is increased.
- To validate the efficacy of the developed Q-learning algorithm for channel prediction, the developed Q-model is investigated using Rayleigh and Rician fading channels.
- Evaluate the beneficial impact of cooperatively integrating the RL based Q-learning algorithm for channel prediction and the derived power allocation scheme for the purpose of multiuser recognition in the power domain MISO-NOMA system.
- The optimized power allocation scheme and the fixed power allocation scheme are both compared when the developed Q-learning scheme is implemented as a channel estimator.

The remainder of this paper is structured as follows. Section 2 describes the system model. Analysis of the optimization problem is presented in Section 3. The optimization framework and procedure are discussed in Section 4. The RL structure is introduced in Section 5. Section 6 discusses the Q-learning algorithm-based channel prediction. The RL-based Q-model architecture and channel estimation algorithm are summarized in Section 7. The simulation environment is described in Section 8, and simulation results are presented in Section 9. Lastly, conclusions are shown in Section 10.

***Notation***: bold lower-case letters denote vectors, bold upper-case letters denote matrices, and lower-case letters denote scalars. The subscript on a lower-case letter $x_{i}$ represent *i*th element of vector x. $E\left(\cdot \right)$ refers to the expectation and (·)^T^ refers to the transpose of the vector. For two real numbers a ≤ b, [a, b] is the set for all real numbers in the range from a to b.

## 2. System Model

### 2.1. Multiuser Environment

In this work, a multiuser environment with a single Base Station (BS) and multiple user devices (UDs) is considered. The BS is supplied with N antennas and all the UDs are supplied with a single antenna. The network is assumed to work with equal length time intervals and each time interval includes one transmission, which contains either uplink or downlink transmissions. The pilot-assisted channel prediction is considered in this work, where pilot symbols can be identified by BS and UDs [15,17]. Each user device initially transmits its pilot symbols to BS via an uplink channel. Then, prior to data transmission, the BS can inspect the pilot symbols and the available network information to facilitate estimating the downlink CSI. The main aim of this work is to model the channel prediction task and to manage the power allocation scheme. We can refer to the matrix of downlink channel coefficients from BS with N antennas to UD *i* as:

(1) $$H_{i}=\left[h_{1i};\;h_{2i};\ldots ;h_{Ni}\right]\;$$

where $h_{ji}$ represents the vector channel parameters from jth antenna at BS to the ith UD, with $j\in \left[1,\;2,\;\ldots ,N\right]$ and $i\in \left[1,\;2,\;\ldots ,M\right]$, where N is the number of antennas at BS and M is the number of users in MISO-NOMA cell. Furthermore, we can denote the data signal transmitted to UD *i* as

(2) $$s_{i}=\left[s_{i1},\;s_{i2},\;\ldots ,s_{iK}\right]\;$$

where *K* is the length of the signal. Then, the matrix of all the UD’s sequences can be expressed as

(3) $$S=\left[s_{1};s_{2};\ldots ;s_{M}\right]$$

The received *k*th signal at *j*th UD can be denoted as:

(4) $$y_{kj}=\sum_{i=1}^{N}h_{ij}s_{ki}+z_{kj}$$

where $z_{kj}$ denotes the AWGN with zero mean and variance $\sigma^{2}$ at *j*th UD through *k*th signal duration. The received *k*th symbol at all UDs is:

(5) $$Y_{k}=\sum_{i=1}^{N}h_{i}s_{ki}+z_{k}$$

where

(6) $$Y_{k}=\left[y_{k1};y_{k2\;};\;\ldots \;;y_{kM}\right]$$

(7) $$z_{k}=\left[z_{k1};\;z_{k2};\;\ldots ;z_{kM}\right]$$

Many of the current works depend on pilot symbols to approximate the uplink channel parameters and then utilize channel reciprocity to realize the prediction of downlink channel weights [15,18]. These schemes for CSI prediction may not be reliable, especially in cases of inadequate channel reciprocity owing to hardware constraints. Furthermore, this kind of estimator may introduce estimation errors in case the uplink and downlink channel parameters are not stationary within a certain transmission.

In the developed Q-learning procedure, we plan to get assistance from the pilot symbols, and network information to explicitly predict the downlink channel parameters. The set of estimated channel coefficients among BS and *M* UDs can be indicated as

(8) $$\hat{H}=\left[{\hat{H}}_{1};\;{\hat{H}}_{2};\ldots ;{\hat{H}}_{M}\right]$$

where ${\hat{H}}_{i}$ is the predicted matrix channel coefficients between BS that contains *N* antennas and *i*th UD, and can be expressed as follows:

(9) $${\hat{H}}_{i}=\left[{\hat{h}}_{1i};\;{\hat{h}}_{2i};\ldots ;{\hat{h}}_{Ni}\right]$$

where ${\hat{h}}_{ji}$ represents the predicted channel parameters between jth antenna at BS and the *i*th UD.

### 2.2. MISO-NOMA Environment

The fundamental idea of NOMA is to achieve non-orthogonal resource allocation between users while increasing the processing at the receiver side [19]. With non-orthogonal resource allocation, NOMA can attain massive connectivity and accomplish higher spectral efficiency. Existing research on the NOMA system mainly focuses on the code domain and power domain. In the code domain NOMA, distinct spread-spectrum codes are designated to different users and then multiplexed over the same time-frequency resource block. In the power domain NOMA (PD-NOMA) [19], the transmitter superimposes signals with different power levels to be sent to several users on the shared spectrum. At the receiver, each user can decode his own desired signal by means of successive interference cancellation (SIC).

**Figure 1:**
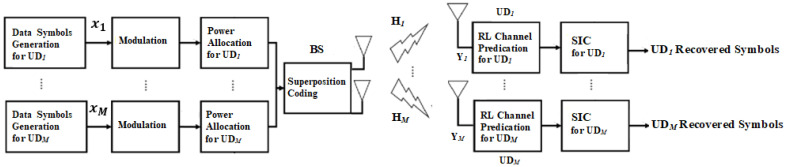
MISO-NOMA system basic Structure based RL channel prediction.

In this subsection, the downlink MISO-NOMA system is explored where user devices and BS are linked by different fading channels. NOMA cell is assumed where one BS with two antennas is implemented to assist user devices (UDs), and each device terminal has one antenna. In PD-NOMA [19], user devices receive the superimposed signal sent from BS which involves target and interfering signals sent through the same resources. Thus, combining different signals supported by unique power portions is critical to distinguish signals and strengthen the successive interference cancellation (SIC) technique. The system structure for the basic components implemented in the examined MISO-NOMA system is shown in Figure 1.

In our observed MISO-NOMA cell, three user devices are considered in the cell, and the examined user devices are identified corresponding to their fading channels and the distances from BS. Fading channels with Rayleigh distribution are adopted to characterize the channel model for every user. The user terminal at the boundary of the cell is realized as a far user, while the nearest user equipment is designated as a near user terminal. The examined cell contains three user devices and the fading path can be distinguished for every user as follows [3]: $h_{n}\sim \left(0,d_{n}^{-k}\right)$ for near users, $h_{m}\sim \left(0,d_{m}^{-k}\right)$ for the middle user, and $h_{f}\sim \left(0,d_{f}^{-k}\right)$ for the user at the edge of the cell, where $h_{i}$ implies a vector represents the fading path coefficients among BS and user *i*. Path loss exponent is represented by *k*, and AWGN is considered with noise power indicated as $\sigma^{2}$. In terms of channel gains, the relation between user devices can be indicated as ${\left|h_{n}\right|}^{2}>{\left|h_{m}\right|}^{2}>{\left|h_{f}\right|}^{2}$ [20] and overall power transmitted from BS to all users in the cell is labelled as $P_{t}$. Every user device contains a receiving element that can activate the SIC process to get rid of signals related to other devices with bad channel environments. In contrast, signals related to user devices with good link conditions may not be separated and regarded as interference. According to the aforementioned assumptions, the superposition-coded signal x sent from BS can be stated as follows [3,17]:

(10) $$x=\sqrt{P_{t}}\left(\sqrt{\eta_{n}}x_{n}+\sqrt{\eta_{m}}x_{m}+\sqrt{\eta_{f}}x_{f}\right)$$

where $\eta_{f}$, $\eta_{m}$ and $\eta_{n}$ represent power factors given for a far device, middle device, and near device separately. Furthermore, $x_{f}$, $x_{m}$ and $x_{n}\;$ refer to the signal vectors related to far, middle, and near users respectively. The received downlink signal at a far device in the MISO-NOMA cell can be shown as:

(11) $$y_{f}=xh_{f1}+xh_{f2}+z_{f}$$

where $h_{f1}$ represents the channel coefficients among a far device and the 1st antenna at BS, $h_{f2}$ represents the channel coefficients among the far device and 2nd antenna at BS and $z_{f}$ is AWGN noise component at the far device with mean zero and variance $\sigma^{2}$. The far user is signified by weak link condition, and signal $x_{f}$ is usually given further power percentage by BS where $\eta_{f}>\eta_{m}>\eta_{n}$. The obtained signal at a far device can be formulated as:

(12) $$y_{f}=\sqrt{P_{t}\eta_{f}}x_{f}\left(h_{f1}+h_{f2}\right)+\left(\sqrt{P_{t}\eta_{m}}x_{m}+\sqrt{P_{t}\eta_{n}}x_{n}\right)\left(h_{f1}+h_{f2}\right)+z_{f}$$

The 1st term in (12) implies the target signal for far device and the 2nd term indicates the interference term from other user devices. The possible bit rate for a far device could be shown as [3,21]:

(13) $$R_{f}=\log_{2}\left(1+\frac{{\left|h_{f1}+h_{f2}\right|}^{2}P_{t}\eta_{f}}{{\left|h_{f1}+h_{f2}\right|}^{2}P_{t}\left(\eta_{n}+\eta_{m}\right)+\sigma^{2}}\right)$$

Typically, the near user device has a good link status alongside BS, therefore, a low power factor can be assigned to $x_{n}$, and the near user received signal can be stated as

(14) $$y_{n}=\sqrt{P_{t}\eta_{n}}x_{n}\left(h_{n1}+h_{n2}\right)+\left(\sqrt{P_{t}\eta_{m}}x_{m}+\sqrt{P_{t}\eta_{f}}x_{f}\right)\left(h_{n1}+h_{n2}\right)+z_{n}$$

In Equation (14), the 1st term represents the anticipated signal, and the 2nd term implies interference from other devices. It can be noted from Equation (14), that the interference can be principal since the far user may be assigned a further power percentage. Thus, at a near device, SIC is accomplished, where direct decoding for the far user signal $x_{f}$ is implemented first, then eliminated from the aggregate signal. After that, the middle device signal $x_{m}$ is decoded and gets rid of it from the resultant signal and the possible rate for a near user $R_{n}$ can be shown as:

(15) $$R_{n}=\log_{2}\left(1+\frac{{\left|h_{n1}+h_{n2}\right|}^{2}P_{t}\eta_{n}}{\sigma^{2}}\right)$$

## 3. Optimization Problem Characterization

The key objective here is to maximize the sum rates for user devices in the MISO-NOMA cell. Sum rate maximization is considered based on optimizing the power coefficients for each user terminal in compliance with the status of the channel between each user and the BS. In downlink MISO-NOMA, the objective function or the sum rates for *M* user devices can be formulated as [3,22]:

(16) $$R_{sum}=\overset{M}{\underset{i=1}{\sum }}\log_{2}\left(1+\frac{{\left|h_{i1}+h_{i2}\right|}^{2}P_{t}\eta_{i}}{{\left|h_{i1}+h_{i2}\right|}^{2}P_{t}\sum_{j=1}^{i-1}\eta_{j}+\sigma^{2}}\right)$$

In the optimization problem, the constraints can be presented as follows:

### 3.1. Power Constraint

The power designated for every user device in the cell is a fraction of the whole power $P_{t}$ sent from BS, therefore the power percentage for each device must conform with [22]:

(17) $$\;\overset{M}{\underset{i=1}{\sum }}\eta_{i}\leq 1$$

where $\eta_{i}$ is the power percentage allocated for the ith user.

### 3.2. QoS Constraints

In our analysis, we consider that all the user devices in the examined MISO-NOMA cell need to satisfy a QoS requirement where the minimum rate $R_{min}$ is required to be realised in the system [22,23], this constraint can be expressed as follows:

(18) $${\mathrm{Log}}_{2}(1+SINR_{i})\geq R_{min}$$

where $SINR_{n}$ is the signal-to-interference plus noise ratio for ith user and $R_{min}$ is the minimum required transmission rate in the examined MISO-NOMA cell. The expression in (18) can be redeveloped as follows [24]:

(19) $${\left|h_{i1}+h_{i2}\right|}^{2}\rho \left(\eta_{i}-(2^{R_{min}}-1)\overset{i-1}{\underset{j=1}{\sum }}\eta_{j}\right)>(2^{R_{min}}-1)$$

where ρ represents the SNR and $\eta_{j}$ is the power percentage given for jth user device.

## 4. Optimization Framework

The main aims in this part include the following: (1) present the objective function and the constraints in a standard form, (2) find a general expression for the 1st and 2nd derivative of the objective function, (3) based on the mathematical analysis and the derived formulas, we can inspect that $\frac{\partial^{2}R_{Sum}}{\partial \eta_{i}{}^{2}}$ is a negative function, which validates that the objective function is a concave with distinctive global maximum, and (4) finally, we deduce the optimal power factors for each user based on applying the Lagrange function and the KKT necessary conditions.

On the basis of the objective function in (16) and the constraints in (17) & (19) and the fact that there are two antennas at the BS and one antenna at each user terminal, the standard optimization problem can be generally reformulated as follows [24,25]:

(20) $$\underset{\eta }{\max}\;R_{sum}=\overset{M}{\underset{i=1}{\sum }}\log_{2}\left(\frac{{\left|h_{i1}+h_{i2}\right|}^{2}P_{t}\sum_{j=1}^{i-1}\eta_{j}+\sigma^{2}+{\left|h_{i1}+h_{i2}\right|}^{2}P_{t}\eta_{i}}{{\left|h_{i1}+h_{i2}\right|}^{2}P_{t}\sum_{j=1}^{i-1}\eta_{j}+\sigma^{2}}\right)\;$$

such that

$$\overset{M}{\underset{j=1}{\sum }}\eta_{j}\leq 1\;\;(2^{R_{min}}-1)-\rho {\left|h_{i1}+h_{i2}\right|}^{2}\left(\eta_{i}-(2^{R_{min}}-1)\overset{i-1}{\underset{j=1}{\sum }}\eta_{j}\right)\leq 0\;\eta_{i}\geq 0\;\;\;\;\;\forall i=1,\;2,\ldots ,M$$

In this part, the power optimisation framework is accomplished with regards to three user devices in the MISO-NOMA cell, therefore, the examined constraints can be represented as shown [25,26]:

(21) $$\;\;\;\;\;\;\;\;\;\;\;\;\;\;\;\;\;\;\;\psi_{1}\left(\eta \right)=\eta_{n}+\eta_{m}+\eta_{f}-1$$

(22) $$\psi_{2}\left(\eta \right)=(2^{R_{min}}-1)-\rho {\left|h_{f1}+h_{f2}\right|}^{2}\left(\eta_{f}-(2^{R_{min}}-1)(\eta_{m}+\eta_{n})\right)$$

(23) $$\psi_{3}\left(\eta \right)=(2^{R_{min}}-1)-\rho {\left|h_{m1}+h_{m2}\right|}^{2}\left(\eta_{m}-(2^{R_{min}}-1)(\eta_{n})\right)$$

Since the constraints $\psi_{1}\left(\eta \right),\;\psi_{2}\left(\eta \right)\;\&\;\psi_{3}\left(\eta \right)\;$ are linear in terms of η, they are considered convex.

Typically, to prove that the objective function $R_{Sum}$ is concave with a distinctive global maximum, we need to find the first derivative $\frac{\partial R_{Sum}}{\partial \eta_{i}}$ and the second derivative $\frac{\partial^{2}R_{Sum}}{\partial \eta_{i}{}^{2}}$ of the objective function [3,24]. The first derivative of the objective function can be deuced in general form as follows [23]:

(24) $$\begin{array}{ll} \frac{\partial R_{Sum}}{\partial \eta_{i}}=\frac{1}{ln2} & \left(\frac{|h_{i1}+h_{i2}{|}^{2}P_{t}}{|h_{i1}+h_{i2}{|}^{2}P_{t}{\sum_{j=1}^{i}\eta }_{j}+\sigma^{2}}\right)\\ & -\frac{1}{ln2}\sum_{k=1}^{M-i}\{\left(\frac{(|h_{(i+k)1}+h_{(i+k)2}{|}^{2}P_{t}{)}^{2}\eta_{i+k}}{\left(|h_{(i+k)1}+h_{(i+k)2}{|}^{2}P_{t}{\sum_{j=1}^{i+k}\eta }_{j}+\sigma^{2}\right)}\right)\\ & \times \left(\frac{1}{\left(|h_{(i+k)1}+h_{(i+k)2}{|}^{2}P_{t}{\sum_{j=1}^{i+k-1}\eta }_{j}+\sigma^{2}\right)}\right)\} \end{array}$$

Similarly, the second derivative of the objective function can be derived in general form as follows [23,24]:

(25) $$\begin{array}{ll} \frac{\partial^{2}R_{Sum}}{\partial {\eta_{i}}^{2}}=-\frac{1}{ln2} & \{\left(\frac{{\left(|h_{i1}+h_{i2}{|}^{2}P_{t}\right)}^{2}}{{\left(|h_{i1}+h_{i2}{|}^{2}P_{t}{\sum_{j=1}^{i}\eta }_{j}+\sigma^{2}\right)}^{2}}\right)\\ & -\sum_{k=1}^{M-i}\{{\left({\left|h_{(i+k)1}+h_{(i+k)2}\right|}^{2}P_{t}\right)}^{3}\eta_{i+k}\\ & \times \left(\frac{\left[2(|h_{(i+k)1}+h_{(i+k)2}{|}^{2}P_{t}\sum_{j=1}^{k+i-1}\eta_{j}+\sigma^{2})+|h_{(i+k)1}+h_{(i+k)2}{|}^{2}P_{t}\eta_{i+k}\right]}{{\left(|h_{(i+k)1}+h_{(i+k)2}{|}^{2}P_{t}{\sum_{j=1}^{i+k}\eta }_{j}+\sigma^{2}\right)}^{2}}\right)\\ & \times \left(\frac{1}{{\left(|h_{(i+k)1}+h_{(i+k)2}{|}^{2}P_{t}{\sum_{j=1}^{i+k-1}\eta }_{j}+\sigma^{2}\right)}^{2}}\right)\}\} \end{array}$$

Based on the above mathematical analysis and the derived formulas, we can inspect that $\frac{\partial^{2}R_{Sum}}{\partial \eta_{i}{}^{2}}$ is a negative function, which verifies that the objective function is a concave with a distinctive global maximum [3,24,27]. To derive the optimal power factors, the Lagrange function and the KKT necessary conditions can be applied [28].

(26) $$L\left(\eta_{n},\eta_{m},\eta_{f},\mu_{1},\mu_{2,},\mu_{3}\right)=R_{Sum}-\mu_{1}\psi_{1}\left(\eta \right)-\mu_{2}\psi_{2}\left(\eta \right)-\mu_{3}\psi_{3}\left(\eta \right)$$

where $\mu_{1}\;,\;\mu_{2},\;$ and $\mu_{3}$ represent Lagrange multipliers for the 3 users’ scenario.

- Optimality conditions can be written as follows [3,24,27]:
  (27) $$\frac{\partial R_{Sum}}{\partial \eta_{n}}-\mu_{1}\frac{\partial \psi_{1}\left(\eta \right)}{\partial \eta_{n}}-\mu_{2}\frac{\partial \psi_{2}\left(\eta \right)}{\partial \eta_{n}}-\mu_{3}\frac{\partial \psi_{3}\left(\eta \right)}{\partial \eta_{n}}=0$$
  (28) $$\frac{\partial R_{Sum}}{\partial \eta_{m}}-\mu_{1}\frac{\partial \psi_{1}\left(\eta \right)}{\partial \eta_{m}}-\mu_{2}\frac{\partial \psi_{2}\left(\eta \right)}{\partial \eta_{m}}-\mu_{3}\frac{\partial \psi_{3}\left(\eta \right)}{\partial \eta_{m}}=0$$
  (29) $$\frac{\partial R_{Sum}}{\partial \eta_{f}}-\mu_{1}\frac{\partial \psi_{1}\left(\eta \right)}{\partial \eta_{f}}-\mu_{2}\frac{\partial \psi_{2}\left(\eta \right)}{\partial \eta_{f}}-\mu_{3}\frac{\partial \psi_{3}\left(\eta \right)}{\partial \eta_{f}}=0$$

Given the fact that ${\left|h_{n}\right|}^{2}>{\left|h_{m}\right|}^{2}>{\left|h_{f}\right|}^{2}$, we can demonstrate that the analyzed constraints are feasible [3] and after a few mathematical manipulations the closed form for the power factors $\eta_{f}$, $\eta_{m}$, and $\eta_{n}$ can be deduced as follows [27]:

(30) $$\eta_{f}=\left(\frac{\left(2^{R_{min}}-1\right)}{2^{R_{min}}}\right)\left(1+\frac{1}{\rho {\left|h_{f1}+h_{f2}\right|}^{2}}\right)$$

(31) $$\eta_{m}=\left(\left(\frac{\left(2^{R_{min}}-1\right)}{2^{R_{min}}}\right)\left(1+\frac{1}{\rho {\left|h_{m1}+h_{m2}\right|}^{2}}\right)-{\left(\frac{2^{R_{min}}-1}{2^{R_{min}}}\right)}^{2}\left(1+\frac{1}{\rho {\left|h_{f1}+h_{f2}\right|}^{2}}\right)\right)$$

(32) $$\eta_{n}=1-\left(\eta_{m}+\eta_{f}\right)\;\eta_{n}=\frac{1}{\left(2^{R_{min}}\right)}\left(\left(\frac{1+\rho {\left|h_{f1}+h_{f2}\right|}^{2}}{(2^{R_{min}})\rho {\left|h_{f1}+h_{f2}\right|}^{2}}\right)+\left(\frac{\left(2^{R_{min}}-1\right)}{\rho {\left|h_{m1}+h_{m2}\right|}^{2}}-\frac{1}{\rho {\left|h_{f1}+h_{f2}\right|}^{2}}\right)\right)$$

## 5. Reinforcement Learning Framework

Typically, RL is developed on the basis of a Markov Decision Process (MDP) design, that contains basic elements [29,30]: a state space ‘***S***’, which is the set of states or observations in the environment and these states can be observed by the agent. An action space ‘***A***’, which is the set of actions that can be selected by the agent at each state. An instantaneous reward ‘***R***’, which is the direct reward that is given to the agent after selecting an action a∈A to transfer to a state s∈S . Policy ‘***P***’ represents the mapping criteria to move from the current observed state to a new state based on the action that will be taken by an agent. Another important element in the RL process is the State-action value function $Q\left(s,a\right)$, which is formally described as the expectation or the average of cumulative discounted rewards when an action **a** ∈ ***A*** is selected by an agent in the state **s** ∈ ***S*** when a certain policy is considered. Furthermore, RL can be considered a method of understanding the agent’s interaction in a stochastic environment by successively selecting actions during a sequence of time periods. Therefore, the main aim of reinforcement learning is to train an agent to carry out a certain task within an uncertain environment [30].

The interaction between the agent and the environment can be described as follows: at each time period, the agent can recognize the observations or states in the environment, and based on the current observation, the agent can identify and carry out a specific action. Then, an immediate reward will be sent from the environment to the agent. The reward is a measure of how effective the action is, when the agent performs a certain action to achieve a specific goal [31]. Basically, at each learning time interval, the RL agent interacts with the environment by following a particular policy that controls the transition between state space to action space.

**Figure 2:**
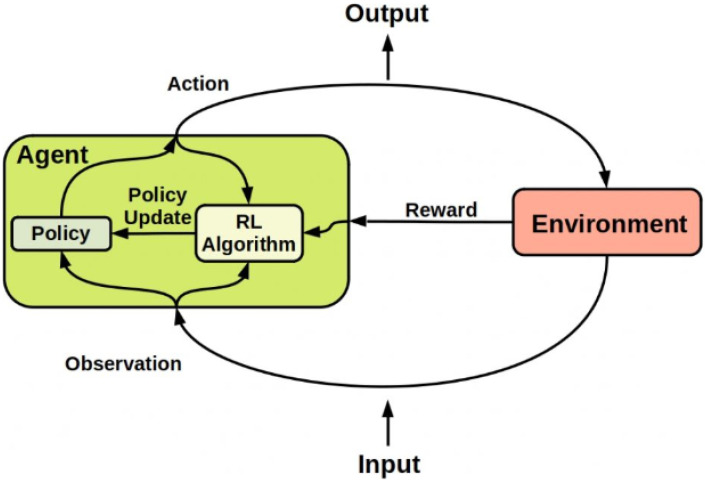
Reinforcement Learning Framework.

Based on the aforementioned discussion and as shown in Figure 2, the RL agent can be essentially represented by two elements: a policy and a learning algorithm [32]. The policy is the mapping criterion that chooses actions on the basis of the observations or status observed in the environment. Usually, the policy can be represented as a function with tunable parameters, such as DNN, while the learning algorithm constantly improves the parameters of the policy based on observations, actions, and rewards [33]. In general, the objective of the learning algorithm is to realize the best possible policy that can maximize the expected cumulative long-term reward received during the task.

## 6. Channel Estimation Based Q-Learning Algorithm

In the considered channel prediction scheme, it is assumed that the action spaces are discrete, therefore, we manage to use an RL-based Q-learning procedure as one of the candidates of RL schemes for parameters update in our examined cell [34,35]. The Q-learning algorithm is categorized as a model-free, and off-policy reinforcement learning procedure, also a Q-learning agent is characterized as a value-based RL agent that has the role of updating a specific critic value function to enhance the future rewards. At a certain state, the agent can inspect and select the action for which the expected reward is maximized. In this section, RL based Q-learning is employed for channel prediction tasks in the MISO-NOMA cells where pilot symbols are also adopted to assist in the channel estimation process [36]. Therefore, it is assumed that there is coordination between BS and user devices such that the pilot symbols can be recognized at the BS and user terminals. In our work, we have considered the BS as the Q-learning agent, and we assume that the BS will start estimating the channel parameters for each user after user devices complete sending the pilot signals [37]. Therefore, in our developed RL based Q-learning algorithm can be utilized to estimate the CSI after the BS receives the pilot signals.

The scenario for the channel prediction process based on the developed Q-learning model can be outlined in this way [38]. Firstly, at the start of each transmission time slot, user devices can send pilot symbols to BS across the uplink channel. Secondly, on the basis of the developed RL based Q-learning algorithm and availability of network information such as user’s distance and path loss, BS (agent) can predict the downlink CSI for user devices. Thirdly, BS will generate the superposition coding signal and performs downlink data transmission. Finally, the receiver of each user terminal will receive the downlink transmitted data and the estimated channel parameters based on Q-learning algorithm will be utilized to decode the desired signal. In addition, each user device can feedback the signal-to-interference plus noise ratio (SINR) or the achieved rate to the BS to enhance the detection process.

In this study, the main objective of the developed RL based Q-learning algorithm is to maximize the downlink sum rate and reduce the estimation loss. Instead of estimating the received signal, we primarily concentrate on incorporating the developed Q-learning model in the NOMA system for the purpose of channel estimation [39]. The RL-based Q-agent is designed to estimate the channel parameters by interacting with the environment, hence strict orthogonal pilot symbols are not required as shown in the standard procedures. Throughout the learning iteration, the Q-learning agent decides on the action that can enhance the approximated state-action value function $Q\left(s,a\right)$ therefore, the expected long-term reward can be also maximized in the neural networks. It is worth mentioning that when increasing the number of learning iterations, updating Q-values becomes more sufficient, and an improved channel approximation and sum rate reward can also be achieved [34,36,40].

In the proposed Q-learning scheme, the sum rate is presented at the learning time interval *t* as $R_{t}$, hence, the instantaneous sum rate at time instant *t* can be shown as follows [15,34]

(33) $$\;\;\;\;\;\;\;\;\;R_{t}=\overset{M}{\underset{i=1}{\sum }}\log\left(1+SINR_{it}\right)$$

where $SINR_{it}$ is the signal-to-interference plus noise ratio of user *i* at time instant *t* and M is the number of users in the MISO-NOMA cell. In this work, the optimum goal of the developed *Q*-learning algorithm is to maximize the total discounted reward $R^{\gamma }$ starting from time instant *t*, which can be denoted as

(34) $$\;\;\;\;\;\;\;\;\;\;\;\;\;\;\;\;R_{t}^{\gamma }=\overset{\infty }{\underset{k=t}{\sum }}\gamma^{k-t}R_{k+1}$$

where $R_{t}^{\gamma }$ is the discounted reward at time slot *t*, and γ is the discount factor. Substituting the sum rate from (33) into (34), the discounted sum rate reward, can be expressed as [41]:

(35) $$R_{t}^{\gamma }=\overset{\infty }{\underset{l=t}{\sum }}\gamma^{l-t}\overset{M}{\underset{i=1}{\sum }}\log\left(1+SINR_{i\left(l+1\right)}\right)$$

As previously stated, the Q-learning agent is the BS, whose aim is to boost the accumulative transmission sum rate. Therefore, two value functions can be inspected while considering the RL maximization problem [34,36,42], the first one is the state value function $V\left(s\right)$

(36) $$\;\;\;\;\;\;\;\;\;\;\;\;\;\;\;\;\;V\left(s\right)=E\left[R^{\gamma }/(S_{t}=s)\right]$$

and the other one is the state-action value function $Q\left(s,a\right)$

(37) $$Q\left(s,a\right)=E\left[R^{\gamma }/(S_{t}=s,A_{t}=a\right)]$$

where E denotes the expected value given that the agent follows a certain policy within the applied procedure. Due to unspecified transition probabilities and limited observed states, an optimal policy is difficult to achieve. Therefore, the *Q*-learning procedure is developed to approximately achieve the best possible policy. In the developed *Q*-learning procedure, the state-action value function $Q\left(s,a\right)$ values are learned via trial and error and are updated according to the following formula [15,34,36,42]:

(38) $$Q\left(s,a\right)\leftarrow \left(1-\alpha \right)Q\left(s,a\right)+\alpha \left[R\left(s,a\right)+\gamma \underset{a^{\prime }\in A}{\max}Q\left(s^{\prime },a^{\prime }\right)\right]\;$$

where α is the learning rate, $s^{\prime }$ denotes the new state, and $a^{\prime }$ is the new action that will be considered by the agent from the action space A to maximize the new state-action value function $Q\left(s^{\prime },a^{\prime }\right)$.

## 7. Q-Learning Network Architecture

**Figure 3:**
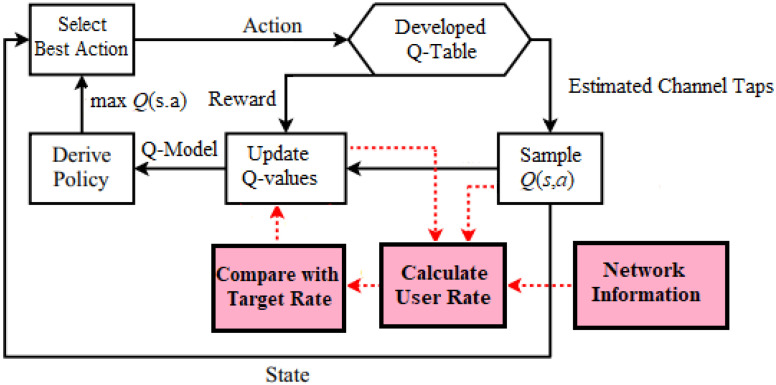
The architecture of the proposed Channel prediction scheme based developed Q-Learning algorithm.

Basically, in data transmission, the frame transmitted includes data and pilot symbols. It is supposed that the implemented channel model is stationary throughout one frame transmission of data and pilot signals and the channel parameters are varying from one frame to another. The basic architecture of the channel prediction scenario based on the developed *Q*-learning procedure employed in our examined network is illustrated in Figure 3, which primarily consists of several stages [17,43].

In the first stage, initial channel parameters will be created based on a distinct Rayleigh channel models. In the second stage, we initialize the ***Q*** table and initialize the reward matrix ***R*** with zero values. The signal-to-interference plus noise ratio (SINR), and the minimum required rate $R_{t}$, can be calculated for every user device in the MISO-NOMA cell with the aid of the availability of the network information such as the initial assigned power percentage for each user terminal, and the entire power transmitted from BS $P_{T}$. Primarily, the Q-values can be adjusted based on the difference between the assigned target rate $R_{T}$ and the initial generated user rate for each device. In the third stage, the best action will be explored and implemented by the ***Q*** agent, and then updating the values for the *Q*-table that represent the observation action pair $Q\left(s,a\right)$. Furthermore, the values for the reward matrix ***R*** will be dynamically assigned according to the actions executed by the Q-agent.

In the fourth stage, the state action value function $Q\left(s,a\right)$ that represent the values for the Q-table will be modified according to a Q-learning procedure with the aid of the following parameters, the discount factor γ, the assigned immediate reward matrix R, and the learning rate α. Throughout the learning phase, the generated state action values $Q\left(s,a\right)$ will be sampled to calculate the new channel rate and at the same time update the Q-table until the optimum rate or the terminal state is achieved.

### Dataset Preparation

Essentially, path loss and the distance between every user terminal and the BS need to be specified in the dataset to facilitate the random generation of the channel weights for every user device in the examined MISO-NOMA network [43]. In the beginning, pilot symbols are created, transmitted, and identified at the BS and at the receiver of every device. Additionally, power factors for every device in the cell need to be initially assigned. The channel weights for every device in the cell are initialized to set up the Q-table values, and during the algorithm iterations, the Q-values are modified according to a Q-learning procedure [34,35,36].

Throughout the learning process and for the sake of updating the *Q*-table, the discount factor γ, learning rate α, the target rate $R_{T}$, current state, and the terminal state should be identified. In our developed Q-learning algorithm, the Q-agent will choose the next state at random and set it as the next state, then the Q-learning agent will inspect all possible actions available to move to the next state. Next, the Q-learning agent will carefully identify the best action a, that satisfies the maximum value for $Q\left(s,a\right)$ to move to the new state. After moving to the new state, a reward value will be assigned to the agent as a measure of how successful this transition was in order to move to the new state [44]. During the update phase, we compute ΔQ, which represents the difference between the new generated value function and the preceding value function of $Q\left(s,a\right)$. Then, update the resultant $Q\left(s,a\right)$ value in the *Q*-table according to the following formula.

(39) $$Q\left(s,a\right)=Q\left(s,a\right)+\alpha \cdot \Delta Q$$

Based on the updated Q-values in Q-table and the updated channel gain, a new achieved rate can be calculated and compared to the target rate for each user device in the cell. In the developed Q-learning algorithm, once the optimum rate or the terminal state is reached, the developed Q-matrix will be employed to compose the channel taps for each user device. The developed Q-learning procedure for channel approximation can be summarized as presented in Algorithm 1.
**Algorithm 1:** Developed *Q*-learning Channel Prediction Structure. Initialize the Q table values and initialize the reward matrix ***R*** with zeroes.**Inputs** 2. Number of Iterations and the size for the channel parameters for every user device.3. Initial distance $“d_{i}”$ of every user device from the BS.4. Path loss parameter “ϑ”.5. Design random pilot symbols.6.Initialize the random channel parameters for each user $“h_{ij}”$ based on fading model, $j\in \left[1,\;2,\;\ldots ,N\right]$ and $i\in \left[1,\;2,\;\ldots ,M\right]$. N is the number of antennas at BS and M is the number of devices in the cell.7. Designate the power percentage $“\eta_{i}”$ for each user.8.Determine system bandwidth “B”, Total transmit power $“P_{T}”$, and noise spectral density $“N_{o}”$9. Assign the desired channel parameters $“h_{id}”$ and the target rate $“R_{T}”$**Procedure** 10.Based on the channel gain ${\left|h_{ij}\right|}^{2}$, total transmit power $“P_{T}”$, and initial power factor for each user $“\eta_{i}”$, signal to interference noise ratio $“SINR_{i}”$, minimum required rate $“R_{i}”$ can be calculated for each device.11.At each iteration, compare the initial generated rate $“R_{i}”$ with the target rate $“R_{T}”$.12.Update the values for the ***Q***-table that represent the current state and action pair $Q\left(s,a\right)$.**Q-algorithm** 13.identify discount factor “γ”, learning rate “α”, the current state, and the terminal state.14. Choose the next state at random and set it as the next new state.15. Inspect all possible actions $“a_{i}”$ to move to the new state.16.Select the best action $a_{i}\in A$, which satisfies the maximum value for the ***Q***-value function argmax $Q\left(s,a\right)$ to move to the new state.17Identify the immediate Reward “R”, based on the action implemented to move to the new state.18.Based on the following: (1) maximum Q-value $Q\left(s,a\right)$ obtained in (16), (2) the corresponding reward “R”, (3) the discount factor “γ”, then $Q\left(s,a\right)$ can be updated based on bellman’s equation$Q\left(s,a\right)\leftarrow R+\gamma \;\mathrm{argmax}\;Q\left(s,a\right)$ **Outputs** 19.Based on the updated $Q\left(s,a\right)$ values in ***Q***-table, the channel coefficients $“h_{ij}”$ and channel gain ${\left|h_{ij}\right|}^{2}$ can be updated and a new user rate can be calculated and compared to the target rate $“R_{T}”$.20.Compute the difference “ΔQ” between the updated value function $Q_{new}\left(s,a\right)$ and the previous $Q\left(s,a\right)$.21.Based on $(20),\;Q\left(s,a\right)$ value in the ***Q***-table can be further updated according to $Q\left(s,a\right)\leftarrow Q\left(s,a\right)+\alpha \cdot \Delta Q$22. Check whether the terminal state has been reached or the episode has been completed.23. Compose predicted channel taps ${\hat{h}}_{i}$

## 8. Simulation Environment

Characterization of the simulation parameters and settings is discussed in this section. The examined downlink MISO-NOMA system contains three distinct user devices and one BS in which the BS is supplied with two antennas and every user device in the cell is provided with a single antenna. In the examined NOMA structure, the modulated signals in downlink transmission are superimposed and transferred by BS to user devices via independent Rayleigh or Rician fading channels that are influenced by AWGN with noise power density assigned as $N_{0}=-174$ dBm/Hz and the path loss is set to 3.5. MATLAB software is utilized as a simulation tool to satisfy the following aims, (1) inspect, characterize, and evaluate the performance of the developed RL based Q-learning algorithm when implemented as a channel estimator in the considered MISO-NOMA system, (2) investigate the reliability of incorporating the developed Q-algorithm as channel estimator scheme with the optimized power scheme in the examined MISO-NOMA network, and performance metrics are considered to explore the impact of this integration. (3) optimized power allocation scheme and fixed power allocation scheme are both compared when the developed Q-learning scheme is utilized as a channel estimator in the cell. Monte-Carlo simulations are performed with $N=10^{5}$ iterations, and at the outset of each iteration, pilot symbols are randomly generated and recognized at the BS and each device. The main simulation parameters are summarized in Table 1.

The presented Simulation figures are generated based on the assumption that the channel coefficients are not available at each user device. Thus, in order to examine the effectuality of the developed RL based Q-learning procedure, and for the sake of comparison, four further simulation environments are established, (1) standard minimum mean square error (MMSE) based channel prediction scheme [45]; (2) DL algorithm based on LSTM network for channel prediction applied in [17], (3) RL based actor-critic procedure for channel prediction applied in [15], (4) the fourth simulation environment is dependent on applying RL based State-Action-Reward-State-Action (SARSA) procedure (Ahsan et al., Mu et al. and Jiang et al.). Throughout the simulations, we point out to MMSE technique as conventional NOMA, to denote that user devices are applying the MMSE technique for predicting the channel state information (CSI) prior to reconstructing the desired signal.

In the simulation environment, NOMA parameters are generated on the basis of the LTE standard [46,47], and channel parameters are created to initially model the Rayleigh fading channels based on the ITU models. In our developed *Q*-learning algorithm, at the end of the training episode, or if the terminal state is reached, the updated $Q\left(s,a\right)$ values in the *Q*-table will be employed as a practical channel coefficients for the user devices. Different power percentages are initially assigned for every user device according to channel gain and based on the existing distance from the BS. Power factors $\eta_{n}$, $\eta_{m}$, and $\eta_{f}$ are specified for near, middle, and far users respectively. In a fixed power allocation setup, we designate $\eta_{f}=0.65$, $\eta_{m}=0.25$, and $\eta_{n}=0.1$. In the optimized power structure (OPS), power factors are allocate d for user devices in proportion to the analytical formula concluded previously for every device in Section 4. In the simulation files, the transmission distance for each user device with respect to BS is assigned as follows: $d_{f}=900\;m$, $d_{m}=400$ m, and $d_{n}=100$ m. Data and pilot symbols are modulated using Quadrature phase shift keying (QPSK) as the modulation format and the applied transferred power is mostly varying from 0 to 30 dBm.

## 9. Simulation Results and Discussion

**Figure 4:**
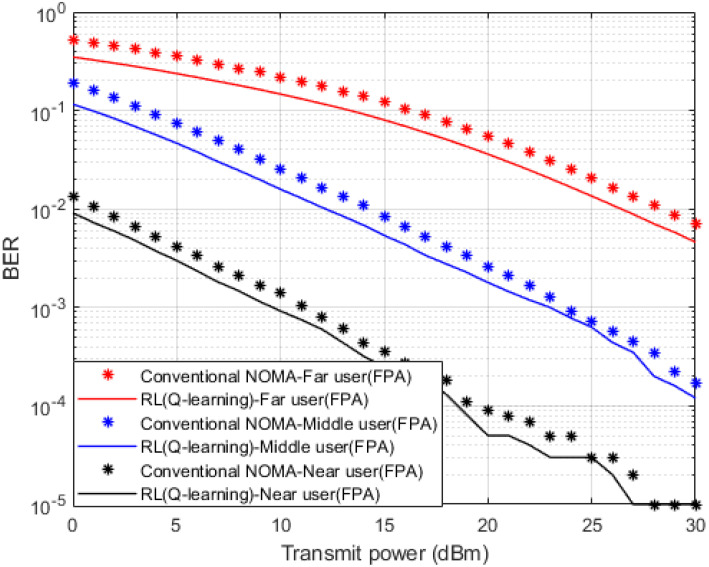
BER vs. power (*Q*-learning & Conventional NOMA (MMSE)).

Simulation outcomes that clarify the comparison between the developed RL based Q-learning algorithm and the conventional NOMA scheme that applies MMSE method to predict the channel coefficients for each device are shown in Figure 4 in terms of BER versus power transmitted. The predicted channel parameters using both schemes are employed for the signal detection for each user device and the simulated results are shown where fixed power allocation (FPA) is considered. When the developed Q-algorithm is applied for channel estimation, each user device in the examined MISO-NOMA cell provides a noticeable improvement in lowering the BER compared to the MMSE procedure. At particular BER values such as 10^−2^, the attained power saving by the Q-learning algorithm is within 2 dBm for far and middle user devices, while a power reduction within 1 dBm is recorded for the near user.

**Figure 5:**
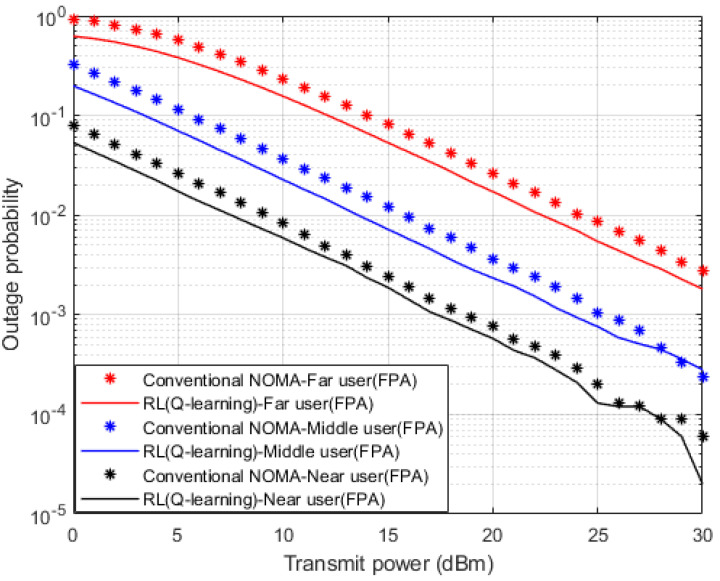
Outage Prob. vs. power (*Q*-learning & Conventional NOMA (MMSE)).

In terms of the outage probability against applied power, Figure 5 illustrates the results for the inspected user devices in the MISO-NOMA cell when the developed Q-learning and standard MMSE are considered as a channel estimator schemes. Far, and middle devices simulation outcomes indicate about 2 dBm enhancement in saving power to realize 10^−2^ outage probability when the developed Q-learning algorithm scenario is applied compared to the MMSE procedure. Similarly, a near user with the developed Q-learning algorithm displays a 1 dBm improvement in power saving with respect to the MMSE scheme. This enhancement in power saving verifies the advantage of the developed Q-model as a channel estimator compared to the MMSE technique.

**Figure 6:**
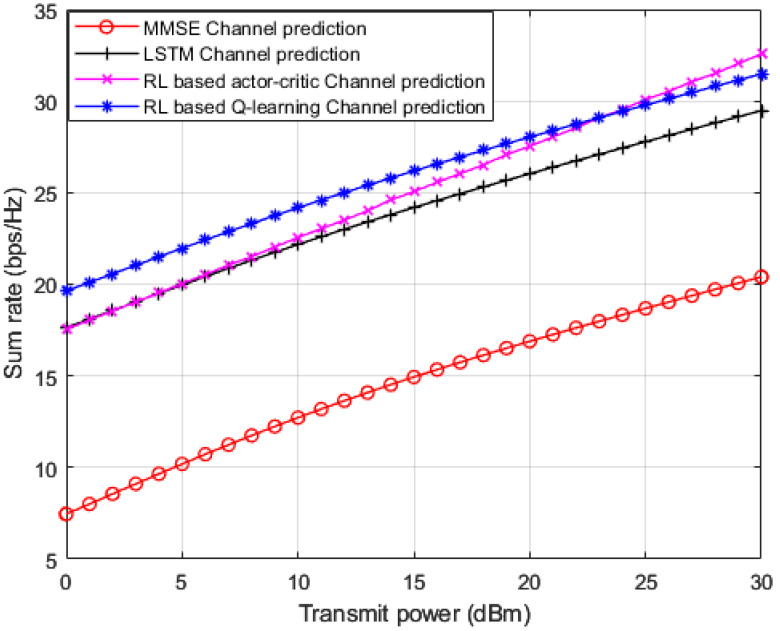
Sum rate vs. power (MMSE, LSTM, RL actor-critic, RL *Q*-learning).

In Figure 6, we implement three baselines for comparisons: (1) standard minimum mean square error (MMSE) based channel prediction scheme [45]; (2) DL algorithm based on LSTM network for channel prediction applied in [17]; and RL based actor-critic procedure for channel prediction applied in [15]. This figure shows simulation results for the sum rate for all the user devices in the MISO-NOMA network versus applied power. Based on the simulation outcomes, it is evidently shown that the developed RL based Q-learning algorithm reveals superiority over standard MMSE procedure by 12 b/s/Hz approximately. Furthermore, the developed Q-learning scheme performs an enhancement over the DL based LSTM procedure presented in [17] by 2 b/s/Hz. For the third benchmark in [15], we generate the simulation environment according to the following: the actor and critic networks are both composed of two hidden layers with 400 and 300 nodes, respectively. The learning rate for actor and critic networks are 10^−4^ and 10^−3^ respectively. The discount factor γ is set to be 0.9 and has a buffer size of 10^5^ [15]. Our developed RL based Q-learning procedure, shows superiority over the RL based actor-critic procedure at low power levels while starting from 23 dBm the actor-critic procedure starts showing some enhancement in terms of sum rates compared to the Q-learning process. These findings can validate that the developed Q-learning algorithm can be a competitive scheme compared to other algorithms that mainly depend on hidden layers to predict channel parameters.

**Figure 7:**
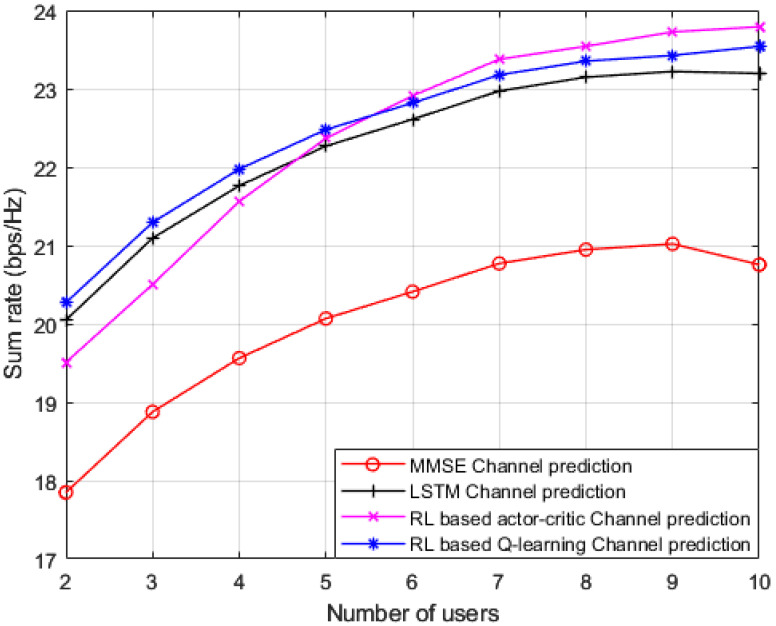
Sum rate vs. number of users (MMSE, LSTM, RL actor-critic, RL *Q*-learning).

Simulation outcomes for the sum rate against different number of users in the applied MISO-NOMA cell are illustrated in Figure 7, where the reference power is chosen to be 1 dBm. In addition to our proposed Q-learning algorithm, three distinct channel prediction methods are investigated as a benchmark comparison: (1) standard minimum mean square error (MMSE) based channel prediction scheme [45]; (2) DL algorithm based on LSTM network for channel prediction applied in [17]; and RL based actor-critic technique for channel estimation applied in [15]. As revealed from the results, our developed RL based Q-learning algorithm can achieve a substantial greater sum rate with respect to standard MMSE procedure, by at least 2 b/s/Hz. It can be observed that as the number of user devices in the cell is increasing, the suggested RL based Q-learning algorithm still shows dominance in accomplishing higher rates with respect to MMSE and DL based LSTM channel estimation methods. Similar to Figure 6, the RL actor-critic procedure applied in [15] is created in our MISO-NOMA environment with the following parameters: the actor and critic networks are both composed of two hidden layers with 400 and 300 nodes, respectively. The learning rate for actor and critic networks are 10^−4^ and 10^−3^ respectively. The discount factor γ is set to be 0.9 and has a buffer size of 10^5^ [15]. As shown in the results, the developed Q-learning scheme is showing an advantage over the actor-critic scheme with up to 6 users in the cell. Then, the hidden layers feature in the actor-critic procedure starts producing some sort of improvement in the sum rates compared to the Q-learning algorithm while the number of user terminals in the cell is increasing. Overall, these outcomes reveal that dependability can be assured by the suggested Q-learning algorithm even when the user devices in the cell are increased. In addition, it is worth saying that while increasing the user devices in the system, the interference will also grow up, thus the sum rate could be degraded.

**Figure 8:**
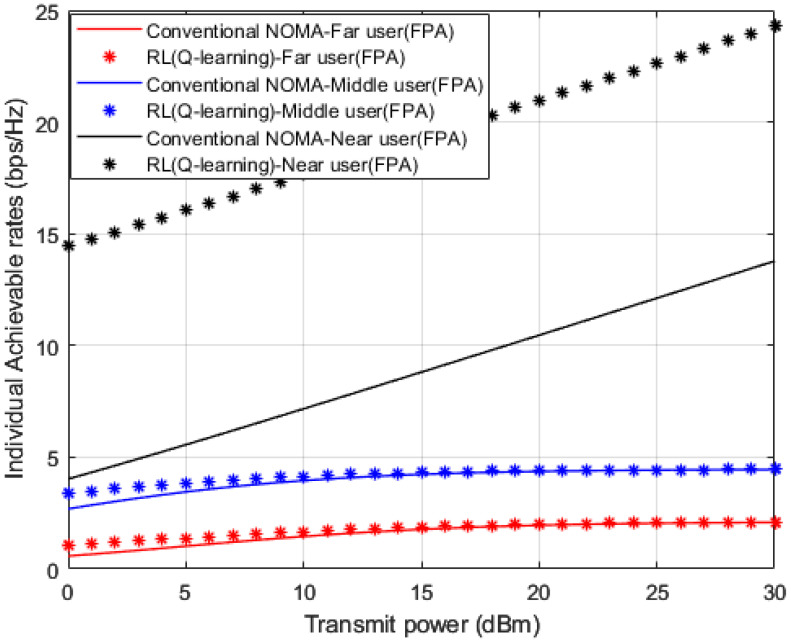
Individual rate vs. power (*Q*-learning, Conventional NOMA (MMSE)).

Figure 8 illustrates simulation outcomes for the achievable capacity for every device in the examined MISO-NOMA system when both the developed Q-learning algorithm and MMSE channel estimation procedures are implemented. The attained rate for near devices reveals substantial improvement by 10 b/s/Hz over far and middle users’ rates. The superiority of the near user in terms of the achievable rate is anticipated, due to the stable channel situation for the near user compared to other devices in the system. Additionally, the suggested Q-learning algorithm still can deliver few visible improvements compared to the MMSE technique for far and middle users’ environments, this slight improvement is associated with the interference and inadequate link conditions for far and middle devices.

**Figure 9:**
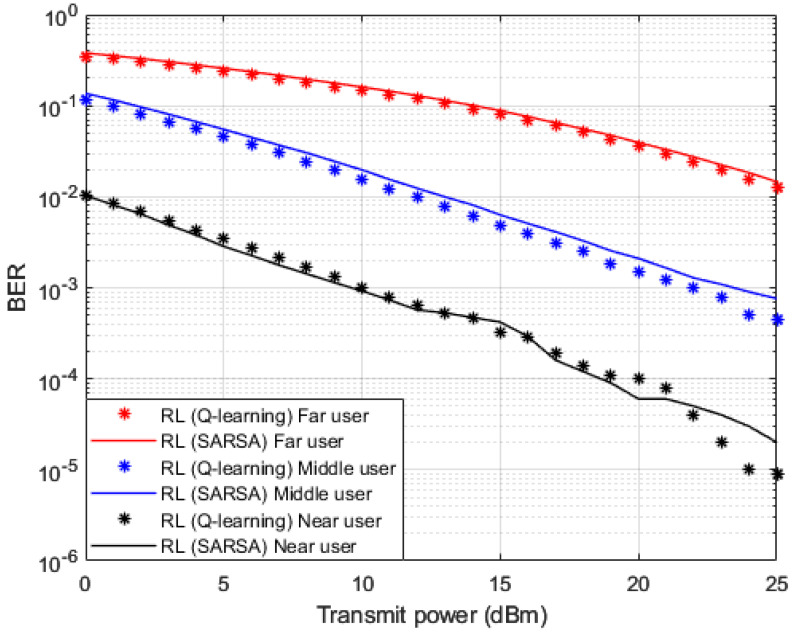
BER vs. power (*Q*-learning, SARSA).

**Figure 10:**
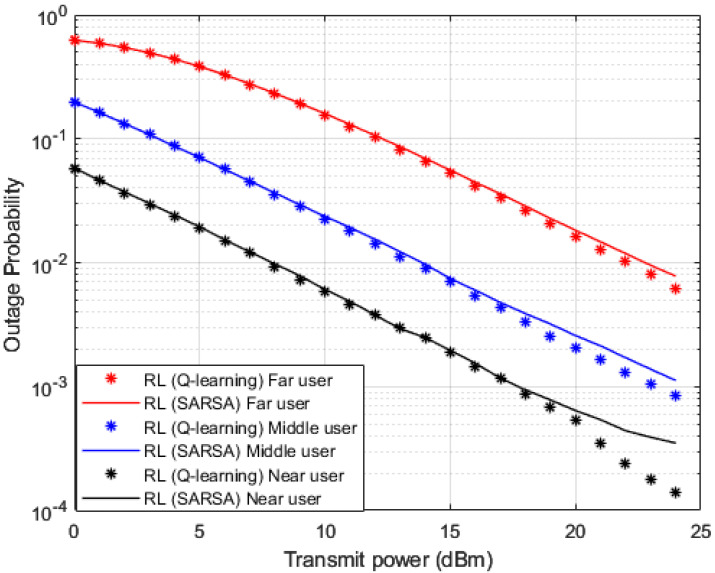
Outage Prob. vs. power (*Q*-learning, SARSA).

**Figure 11:**
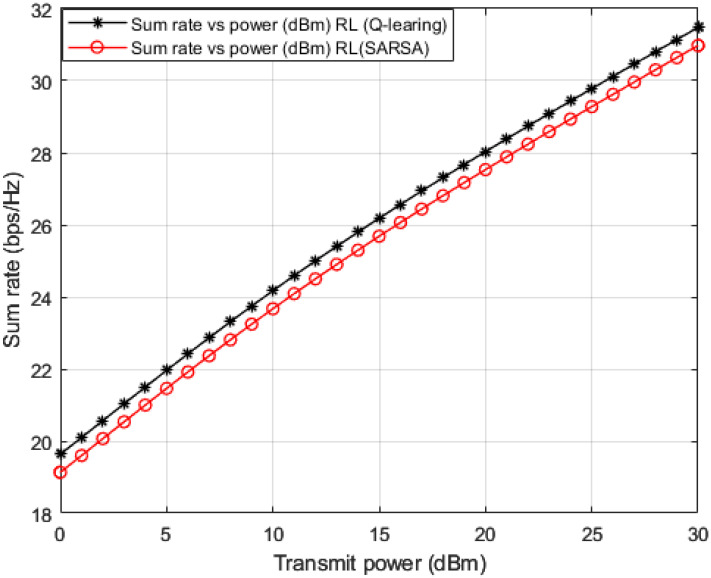
Sum rate vs. power (*Q*-learning, SARSA).

In addition to the three baselines comparisons implemented in Figure 6 and Figure 7, we also create and implement RL based State-Action-Reward-State-Action (SARSA) algorithm [48,49,50] in Figure 9, Figure 10 and Figure 11 for the purpose of more investigations and benchmark comparisons. The features and parameters of the SARSA algorithm are adapted in order that the SARSA procedure can be used as a channel estimator and compare the results of SARSA algorithm with the results obtained based on our developed Q-learning algorithm.

The Q-learning algorithm and SARSA algorithm are two efficient RL algorithms, they are both table-based procedures with a Q-table to record equivalent Q-values of each state-action pair. However, when the size of state space increases, it will need a considerable amount of memory. Similar to the Q-learning algorithm, the SARSA algorithm also has exploration and exploitation processes, and it also needs a Q-table to record $Q\left(s_{t},a_{t}\right)$ value corresponding to state $s_{t}$ and action $a_{t}$. Differently, the running steps of the SARSA algorithm are as follows. First, according to the action selection scheme, the gent at the current state $s_{t}$, will select the action $a_{t}$. Then, the agent gets an immediate reward R based on the corresponding $Q\left(s_{t},a_{t}\right)$ value. Finally, $s_{t}$ will transfer to $s_{t+1}$ and the agent will choose the next action $a_{t+1}$. Hence, the SARSA algorithm is a bit different from the Q-learning procedure, where the Q-value in the SARSA method is updated based on the action $a_{t}$ implemented by the agent at the state $s_{t}$. While in the Q-learning algorithm, the action with the greatest Q-value in the next state $s_{t+1}$ is employed to update Q-table.

In Figure 9 and Figure 10, where BER and outage probability metrics are simulated against transmitted power, both our developed Q-learning and SARSA algorithms show comparable performance. However, at high power levels, the suggested Q-learning algorithm shows little improvement compared to the SARSA algorithm, which may be justified that the Q agent deciding the greedy action, which is the action that provides the maximum Q-value for the state. More investigations for the comparison between SARSA and the developed Q-learning algorithms are shown in Figure 11. Sum rates versus applied power are simulated in Figure 11, and it is noticed that the suggested Q-learning scheme provides an advantage over the SARSA algorithm, and a power saving is recorded by 1–2 dB approximately.

**Figure 12:**
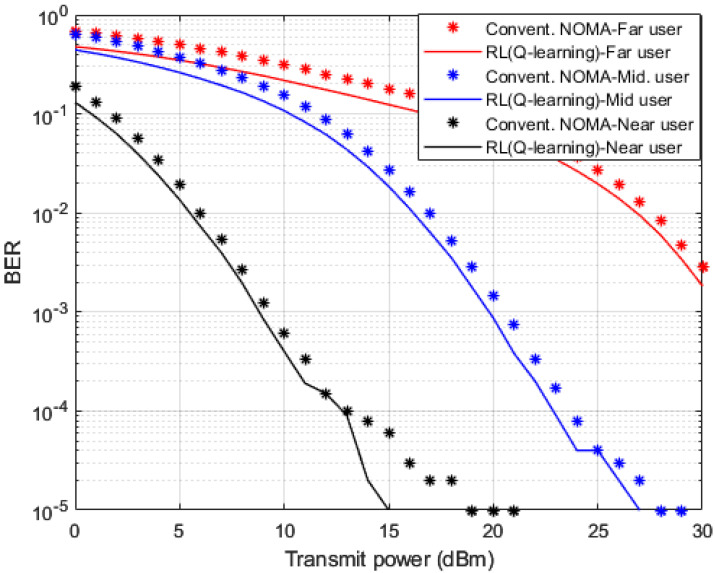
BER vs. Power (*Q*-learning, Conventional NOMA (MMSE)—Rician channel).

The proposed Q-learning method and traditional MMSE technique will be further examined when the Rician channel is applied for the path between BS and each user device. Rician channel is a stochastic model for wireless transmission where the signal reaches the receiver device via various scattered paths. Figure 12, illustrate simulation outcomes for BER against power transmitted when the Rician fading channel is applied. In the Rician simulation environment, we assign parameter *K* = 10, where *K* is described as the fraction of the signal power of the line-of-sight path to the signal power of the remaining scattered components. In addition, maximum doppler shift = 100 and sample rate = 9600 Hz are used. Results for the Rician channel indicate that the Q-learning algorithm still can provide some sort of enhancement in decreasing the BER compared to the MMSE procedure. This slight improvement can be explained by the existence of a line of site component among BS and user terminal which can enhance the work of the MMSE procedure.

**Figure 13:**
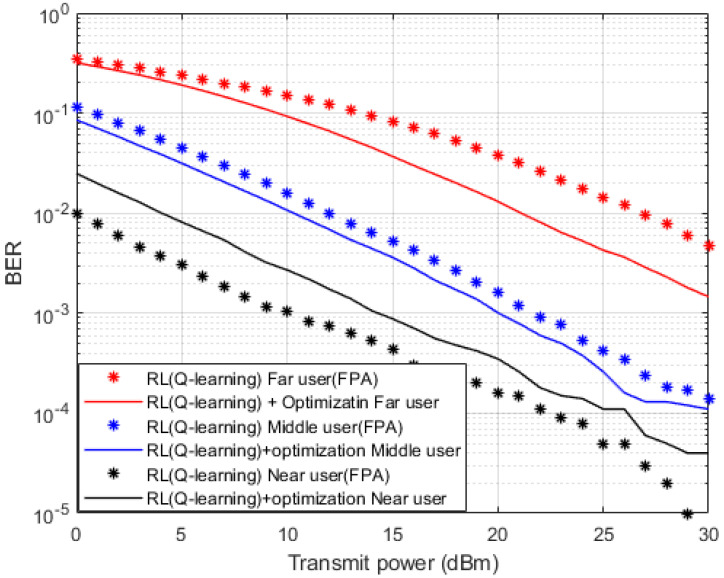
BER vs. Power (*Q*-learning, Optimization, FPA).

In Figure 13, two separate simulation setups are accomplished here to produce these results. In the first setup, the Fixed Power Allocation (FPA) structure is assigned for every user terminal in the MISO-NOMA cell. The second setup depends on the Optimized Power Structure (OPS) applied in accordance with the analytical power scheme that previously concluded in Section 4. FPA or OPS will be applied in conjunction with the suggested Q-learning algorithm as a channel estimator. Simulation outcomes in terms of BER indicate that far and middle users show the dominance of the OPS over the FPA. It can be noted that at specific BER values such as 10^−2^, the achieved power saving by OPS policy is about 5 dBm for the far user, and 1–2 dBm approximately for the middle user. For near user results, the developed Q-learning algorithm jointly with the FPA scheme provide evident improvement in terms of BER over OPS, this could be clarified that for near device scenario, the stable channel condition provides more advantageous for the performance than the assigned power.

**Figure 14:**
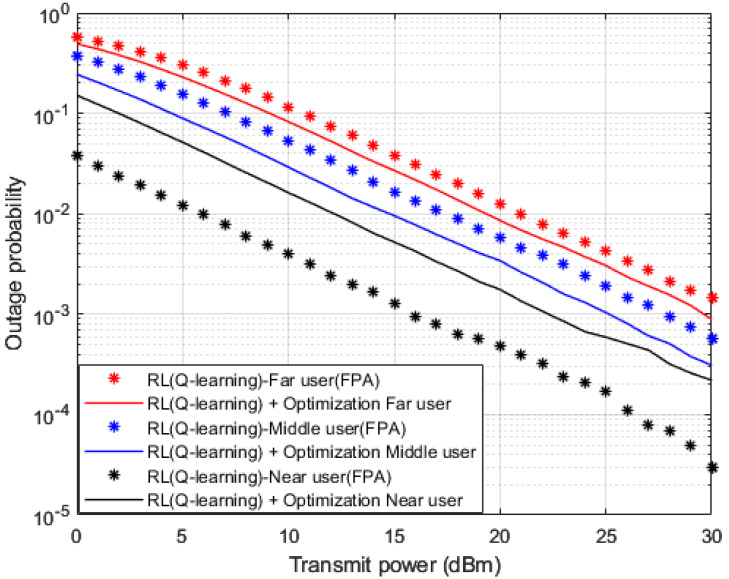
Outage Prob. vs. Power (*Q*-learning, Optimization, FPA).

Outage probability results versus power are shown in Figure 14, where OPS and FPA schemes are also implemented. Both arrangements of OPS and FPA are implemented in conjunction with the proposed Q-learning algorithm as a channel estimator in the MISO-NOMA cell. Both far user and middle user results reveal an improvement in outage probability where a power reduction can be observed within 1–2 dBm when OPS is applied compared to the FPA scheme. On the other hand, near user with a Q-learning algorithm and FPA scenario shows a considerable outage improvement compared to the OPS case. A power reduction within 5 dBm is achieved when the FPA scheme is applied. These findings verify the results obtained for BER in Figure 13, which indicate that the FPA scheme is more adequate for user devices with high channel gains.

**Figure 15:**
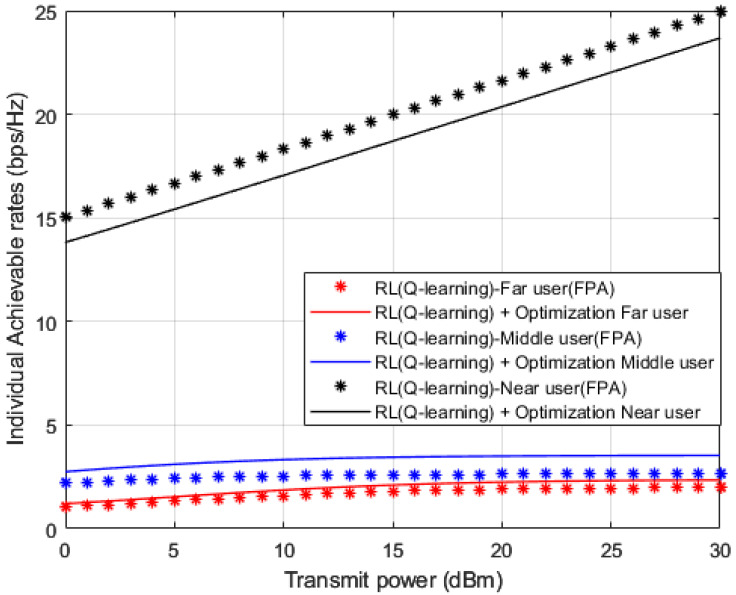
Individual rate vs. Power (*Q*-learning, Optimization, FPA).

In Figure 15, attainable rates for user devices are simulated against power transmitted when OPS and FPA schemes are applied in conjunction with the proposed Q-learning algorithm that is applied as a channel estimator. Results for far and middle devices point out that OPS provides 1 b/s/Hz improvement compared to the FPA scheme. This limited improvement might be clarified where the management of the power allocation for devices is not necessarily sufficient enough to alleviate the influence of interference particularly for far and middle devices that mainly experience unstable links environments. As expected, results for near user device reveal superiority in achieved rate with respect to middle and far devices with at least 10 b/s/Hz. Furthermore, the results for the near user with FPA indicate a noticeable improvement compared to OPS, which validates the results obtained in Figure 13 and Figure 14.

In the end, we can further provide the analysis of the computational complexity as follows: The complexity of the reinforcement learning algorithm mainly depends on the size of the state space and the size of the action space [51]. According to [51], we can approximate the computational complexity of the Q-learning algorithm as $O\left(SAH\right)$ per iteration, where *S* is the number of states, *A* is the number of actions, and *H* is the number of steps per episode. According to the state space and action space defined in our simulation environment, the amount of work per iteration can be approximated as $O\left(NMK\right)$ [51,52], where N represents a number of antennas in BS, M represent a number of user devices in the cell, and K represents the size of channel coefficients. On the other hand, the computational complexity for the benchmark scheme implemented in [15], is described as follows: the sizes of the input layer, the first hidden layer, the second hidden layer, and the output layer for each network implemented in [15] is denoted as I, $h_{1}$, $h_{2}$, and U respectively. Thus, the total number of parameters in each network can be denoted as $\theta =I+h_{1}+h_{2}+U$, therefore, the complexity of this scheme regarding the channel estimation task can be approximated as $O(MN_{A}\left(I+h_{1}+h_{2}+U\right)$ [15], where M represents the number of user terminals and $N_{A}$ represent the number of antennas at BS. According to [53], the corresponding computational complexity for the traditional channel estimation method based MMSE can achieve a relatively low complexity, $O\left(M^{2.37}\right)$ [45,53], but, at the cost of performance degradation. Based on the aforementioned analysis, it can be shown that the complexity of the developed RL based Q-learning algorithm is competitive compared to other procedures.

## 10. Conclusions

In this study, the influence of adopting a developed RL based Q-learning algorithm to distinctly predict the channel parameters for every user device in the MISO-NOMA system is analyzed. In the developed Q-learning algorithm, the Q-model is created on the basis of the initialized channel statistics then updated based on the interaction between the Q-agent and the environment to maximize the received downlink sum rate and minimize the estimation loss. The efficacy of the developed Q-learning procedure is investigated by inspecting the performance of the proposed algorithm against different benchmark channel estimation schemes. The first benchmark scheme is based on standard MMSE procedure, the second approach is applying DL based LSTM network, the third scheme is implementing RL based actor-critic algorithm, and the fourth benchmark scheme is using RL based SARSA algorithm. In addition, the reliability of the proposed Q-learning procedure is validated by analyzing the behavior of the developed Q-learning algorithm in different fading channels. Furthermore, we provided a scenario that explores how the proposed channel prediction method based on Q-learning algorithm and the derived power allocation structure are both cooperatively employed for multiuser recognition in the MISO-NOMA network. Simulation results emphasized that dependability can be ensured by the developed Q-model even when the number of users in the cell is increased. Furthermore, the simulation outcomes in terms of BER, Outage probability, and individual user rate have demonstrated that the developed Q-learning algorithm for channel estimation jointly with an optimized power scheme can both realize consistent performance.

## Figures and Tables

**Table 1:** Summary of Simulation Parameters.

Parameter	Value
Simulation Tool	MATLAB
Modulation type	QPSK
Number of Users	3, [2,3,4,5,6,7,8,9,10]
System Bandwidth *B*	1000 kHz
Fading channel	(Rayleigh, Rician)
Path loss exponent	3.5
Number of Iterations	10^5^
Noise PSD $N_{0}$	−174 dBm/Hz
Learning Rate α	0.1
Discount factor γ	0.9

## Data Availability

Not applicable.

## Abbreviations

The following abbreviations are used in this manuscript:

AWGN	Additive White Gaussian Noise
BER	bit error rate
BS	Base Station
CSI	Channel state information
DL	Deep Learning
DNN	Deep Neural Network
FPA	Fixed Power Allocation
OPS	Optimized Power structure
KKT	Karush-Kuhn–Tucker
LSTM	Long Short-Term Memory
LTE	Long Term Evolution
ML	Machine Learning
MSE	Mean Square Error
MMSE	Minimum Mean Square Error
MUD	Multiuser detection
PD-NOMA	Power Domain Non-Orthogonal Multiple Access
QoS	Quality of Service
SIC	Successive interference cancellation
MISO	Multi-input single-output
SARSA	State-Action-Reward-State-Action
RL	Reinforcement Learning
